# Association between periodontitis and all-cause and cancer mortality: retrospective elderly community cohort study

**DOI:** 10.1186/s12903-020-01156-w

**Published:** 2020-06-09

**Authors:** Ping-Chen Chung, Ta-Chien Chan

**Affiliations:** 1 Department of Dentistry, Puzi Hospital, Ministry of Health and Welfare, Chiayi, Taiwan; 2 Research Center for Humanities and Social Sciences, Academia Sinica, 128 Academia Road, Section 2, Nankang, Taipei, 115 Taiwan; 3 Institute of Public Health, School of Medicine, National Yang-Ming University, Taipei, Taiwan

**Keywords:** Periodontitis, Cancer, Mortality, Smoking

## Abstract

**Background:**

Periodontal infection induces inflammation, which may increase the risk of tumor-promoting effects. The aim of this study was to assess the association between periodontitis and all-cause mortality, and all-cancer and specific cancers’ mortality in a health examination cohort of the elderly in the communities.

**Methods:**

A dataset of health examinations for the elderly with cause of death from 2005 to 2012 was obtained from the Department of Health, Taipei City Government. We enrolled 82,548 study participants with 262,035 visits. A Cox proportional hazards model and Cox frailty model were used for calculating the hazard ratios under different periodontal status by using SAS and Rstudio.

**Results:**

Being male, elderly, having a low education level, and smoking were risk factors for mortality in this retrospective elderly community cohort study. Participants with periodontitis followed across time had significantly higher hazard ratios (HRs) for all-cause mortality and all-cancer mortality (HR = 1.092, 95% confidence interval (CI):1.038 to 1.149, HR = 1.114, 95% CI:1.032 to 1.203, respectively) in the Cox frailty models after adjusting for age, marital status, education level, sex, and smoking status. After adjusting for age and sex, the hazard ratio was 1.185 (95% CI: 1.027 to 1.368) for lung cancer mortality, and 1.340 (95% CI: 1.019 to 1.762) for prostate cancer mortality in the periodontitis group with each visit.

**Conclusions:**

The findings indicated that being male, having a low education level, and daily smoking were risk factors for mortality, and showed mixed evidence that periodontal disease is associated with all-cause, all-cancer and specific-cancer mortality including lung and prostate cancer. We suggest the importance of regular health screening in order to achieve early disease detection and lower mortality risk.

## Background

Periodontitis is a process in which periodontal bacteria [1] and viruses [2] lead to a host immune-inflammatory response in periodontal tissues that causes periodontal pocket formation, attachment loss and bone loss. Numerous studies have pointed out the relationship between periodontitis and systemic diseases such as cardiovascular disease [3], diabetes [4], respiratory disease, especially chronic obstructive pulmonary disease [5], and chronic kidney disease [6]. Furthermore, periodontitis is also associated with several types of cancers such as lung [7], esophageal [8], pancreatic [9], kidney [10], and haematological cancer [11]. Periodontal infection induces inflammation that may increase the risk of tumor-promoting effects [12, 13].

Periodontitis is a serious public health issue throughout the world. In 2010, severe periodontitis was the sixth most prevalent condition globally [14]. Globally, the prevalence of severe periodontitis peaked at age 60 to 64 years old, and the global age-standardized prevalence of severe periodontitis was 9.8% (95% uncertainty interval, 8.2 to 11.4%) in 2017 [15]. The prevalence of periodontitis significantly increased from 11.5% in 1997 to 19.59% in 2013 in Taiwan. The pattern of mean age for periodontitis decreased from 1997 to 2013 (mean age  ± standard deviation: 54.46  ±  14.47 and 45.51  ±  16.58 years old, respectively) [16]. A cross-sectional nationwide survey on periodontal conditions in Taiwan between 2007 and 2008 revealed that the prevalence of a value of 3 or greater on the community periodontal index ((CPI) ≥3), which is used to measure the periodontal status and record bleeding, calculus and pocket depth, among those 65 to 74 years old and above 74 years old was 72.7 and 76.7% respectively [17, 18]. Severe periodontitis is related to poor oral health conditions which can lead to tooth loss, unclear speaking, difficulty chewing and swallowing, poor nutrition, and poor quality of life [19].

Pathogenic microorganisms such as *Porphyromonas gingivalis, Treponema denticola* and *Tannerella forsythia* lead to chronic inflammation and destruction of periodontal soft and hard tissues [20]. The spread of bacteria and inflammatory mediators from the oral cavity can elevate and sustain systemic inflammatory conditions and damage to various organs [9, 21]. The inflammatory reaction directly or indirectly induces cell proliferation and the release of reactive oxygen species and other metabolites that can promote cancer initiation [21, 22].

Thus, the primary goal of periodontal therapy is to arrest the inflammatory disease process. Regardless of age, nonsurgical and surgical periodontal treatments for periodontal disease are effective therapies, removing subgingival biofilm and doing plaque control to maintain periodontal health [23, 24]. The aim of this study was to assess the association between periodontitis and all-cause mortality, all-cancer and specific cancers’ mortality in a health examination cohort of the elderly in the communities.

## Methods

### Study design and population

This was a retrospective cohort study from January 1, 2005 to December 31, 2012, which used a dataset of health examinations for the elderly with age equal to or above 65 years old, performed by Taipei-contracted hospitals and supported by the Department of Health, Taipei City Government in Taiwan. Participation in the annual health examinations was voluntary for senior citizens. The study population received an interview, physician consultation and clinical examination from January 1, 2005 to December 31, 2008.

**Fig. 1:**
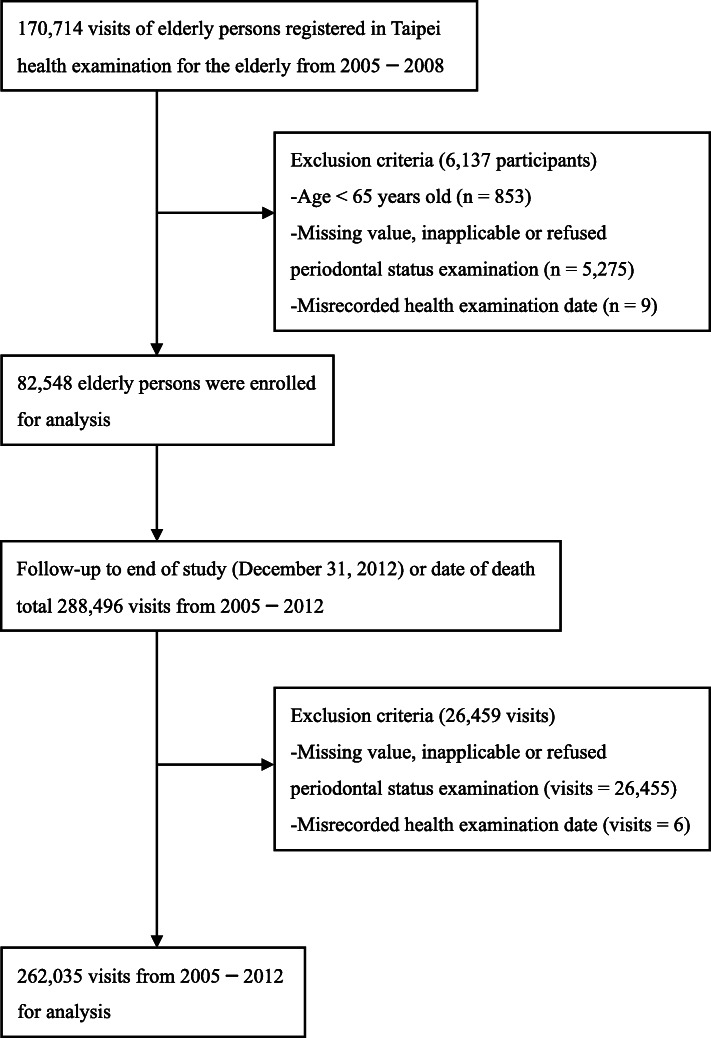
The flow chart of the study sample enrollment and follow-up

Those aged less than 65 years old (*n* = 853), or with a misrecorded examination date (*n* = 9) or missing data on periodontal status *(n* = 5257) at the first visit were excluded. Finally, we enrolled 82,548 study participants for further analyses. The total visits numbered 262,035 as of the end of the study after excluding 26,461 visits with missing data regarding periodontal status *(n* = 26,455) or misrecorded examination dates (*n* = 6) (Fig. 1).

### Assessment and definition of periodontitis

In the oral examination, participants with periodontal status reported as “inapplicable” or “refused” were excluded. If participants’ periodontal status as diagnosed by dentists showed “no obvious abnormalities” then these participants were classified as having healthy periodontium, while participants with “abnormal periodontal status” diagnosis and periodontal tissues described as “tooth mobility” or “periodontitis” by dentists were classified as having periodontitis.

### Outcome definition

The primary endpoint was the date of death, especially death from cancer, or the end of the follow-up period (December 31, 2012). The cause of death was recorded according to the International Classification of Diseases, Ninth Revision (ICD-9: 001–998) or Tenth Revision (ICD-10: A00-Z99) [25].

### Measurement of exposure and potential confounders

The baseline interview collected age, sex, education level (illiterate, 1–6 years of schooling, 7–14 years of schooling, or above 14 years of schooling), marital status (married and living together, vs. others), self-reported smoking status in the past 6 months (smoked every day, smoked some days or socially, or did not smoke), and self-reported intake of two fruits and three dishes of vegetables daily (yes, no). If the participant had a history of diabetes or took long-term medication for controlling diabetes, or the fasting blood glucose report revealed abnormality, then the participant was defined as diabetic. In each oral examination, periodontal status was recorded by dentists.

### Statistical analysis

The proportions of participants with different periodontal status at the baseline were calculated separately by demographic characteristics and health behaviors. Comparisons of baseline characteristics between subgroups according to the periodontal status were made using logistic regression in which the first category in each variable was regarded as the reference group. Kaplan-Meier curves with the log-rank test were employed to demonstrate the differences in survival curves in subgroups of different periodontal status at the baseline. At each time point, Kaplan-Meier survival data included the numbers at risk.

A Cox proportional hazards model [26] and Cox frailty model [27, 28] were used for calculating the hazard ratios of all-cause mortality and all-cause cancer mortality under different periodontal status. A Cox proportional hazards model as a semi-parametric model is a common method for study of time-to-event data [26]. A Cox frailty model is a time-dependent model considering random effects of time. This approach can be used for repeated events for the same individual [29]. In our elderly cohort, a participant might be examined in several years, and the smoking and periodontal status might be different each time. The Cox frailty model is a suitable method of analysis. The coefficients estimated from the frailty models might differ from those of the general Cox model if there is a meaningful contribution of the random term. After deleting participants who had one or more missing covariates regarding education level (*n* = 12,592), marital status (*n* = 1347), and smoking status (*n* = 335) in the baseline data, the Cox proportional hazards model and the Cox frailty model estimated the hazard ratio for all-cause and all-cancer mortality and included age, sex, education level, marital status, smoking status and periodontal status. Due to the low number of each specific cancer to test the association, besides periodontal status, the Cox frailty models of deaths from cancer were only adjusted for age and sex. Hazard ratios and 95% confidence intervals of all-cause, all-cancer and specific cancer mortalities in subgroups are summarized in Table S1. All analyses were two-sided with alpha set at 0.05. We conducted all statistical analyses by using SAS (version 9.4) and RStudio (Version 1.0.153) with packages of survival [30], and ggplot2 [31].

## Results

### Characteristics of the study population

In the baseline, 24,806 participants had periodontitis (30.05%). The mean age of the sample at baseline was 73.59 years, and the slight majority were males (52.15%). More participants had 7–14 years of schooling (43.12%), were not married and living together (72.95%), had not smoked in the past 6 months (90.86%), ate fruits and vegetables daily (77.41%) and were not diagnosed with diabetes (65.8%). The association of periodontal status with demographic and health behaviors is presented in Table 1. The following are significant based on *p* < 0.05. When comparing participants with healthy periodontium to participants with periodontitis, the latter were more likely to be male (32.98%), be illiterate (32.70%), have higher frequency of smoking (smoke daily 39.82%) and have no fruit and vegetable intake daily (33.10%). However, there was no obvious difference (p > 0.05) in the percentages between the healthy periodontium group and periodontitis group by marital status and diabetes diagnosis based on univariate logistic regression. At the end of study, the number of deaths was 11,160 participants, among which about 33.15% had periodontitis.

**Table 1:** Baseline characteristics of elderly participants with different periodontal status in Taipei (2005–2008)

	Total	Healthy periodontium		Periodontitis		
	N	N	(%)	N	(%)	
Age (*n* = 82,548)	73.59 ± 6.54	73.61 ± 6.59		73.54 ± 6.44		
Sex (*n* = 82,548)						
female	39,496	28,890	(73.147)	10,606	(26.853)	
male	43,052	28,852	(67.017)	14,200	(32.983)	*
Education (*n* = 69,956)						
illiterate	4600	3096	(67.304)	1504	(32.696)	
1–6 years	21,330	14,831	(69.531)	6499	(30.469)	*
7–14 years	30,162	21,458	(71.142)	8704	(28.858)	*
above 14 years	13,864	9972	(71.927)	3892	(28.073)	*
Marital status (*n* = 81,201)						
married living together	21,966	15,315	(69.721)	6651	(30.279)	
other	59,235	41,468	(70.006)	17,767	(29.994)	
Smoking status (*n* = 82,213)						
no	74,697	52,781	(70.660)	21,916	(29.340)	
occasionally	4558	2944	(64.590)	1614	(35.410)	*
daily	2958	1780	(60.176)	1178	(39.824)	*
Eat fruits and vegetables (*n* = 81,978)						
no	18,517	12,388	(66.901)	6129	(33.099)	
yes	63,461	44,955	(70.839)	18,506	(29.161)	*
Diabetes (*n* = 82,548)						
no	54,286	38,037	(70.068)	16,249	(29.932)	
yes	28,262	19,705	(69.723)	8557	(30.277)	
All-cause mortality (*n* = 82,548)						
no	71,388	50,282	(70.435)	21,106	(29.565)	
yes	11,160	7460	(66.846)	3700	(33.154)	*
All-cancer mortality (*n* = 82,548)						
no	79,033	55,380	(70.072)	23,653	(29.928)	
yes	3515	2362	(67.200)	1153	(32.802)	*

*: logistic regression *p*-value< 0.05

age: mean ± standard deviation

**Fig. 2:**
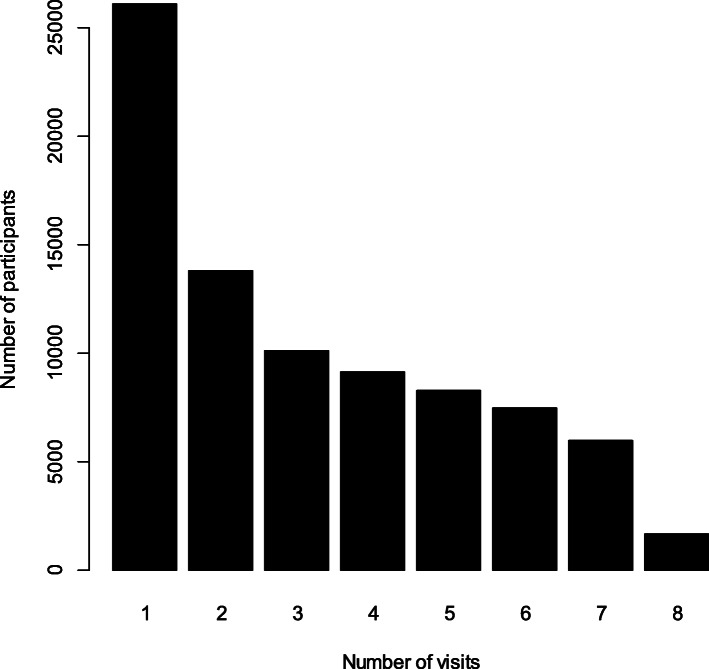
Distribution of elderly health exam frequency at baseline

As of December 31, 2012, among 82,548 participants the average number of visits was 3.17 times (Fig. 2). The maximum of visits was 8 times, involving 1666 participants (2.02%).

### Association between periodontal status and risk of all-cause mortality and all-cancer mortality

**Fig. 3:**
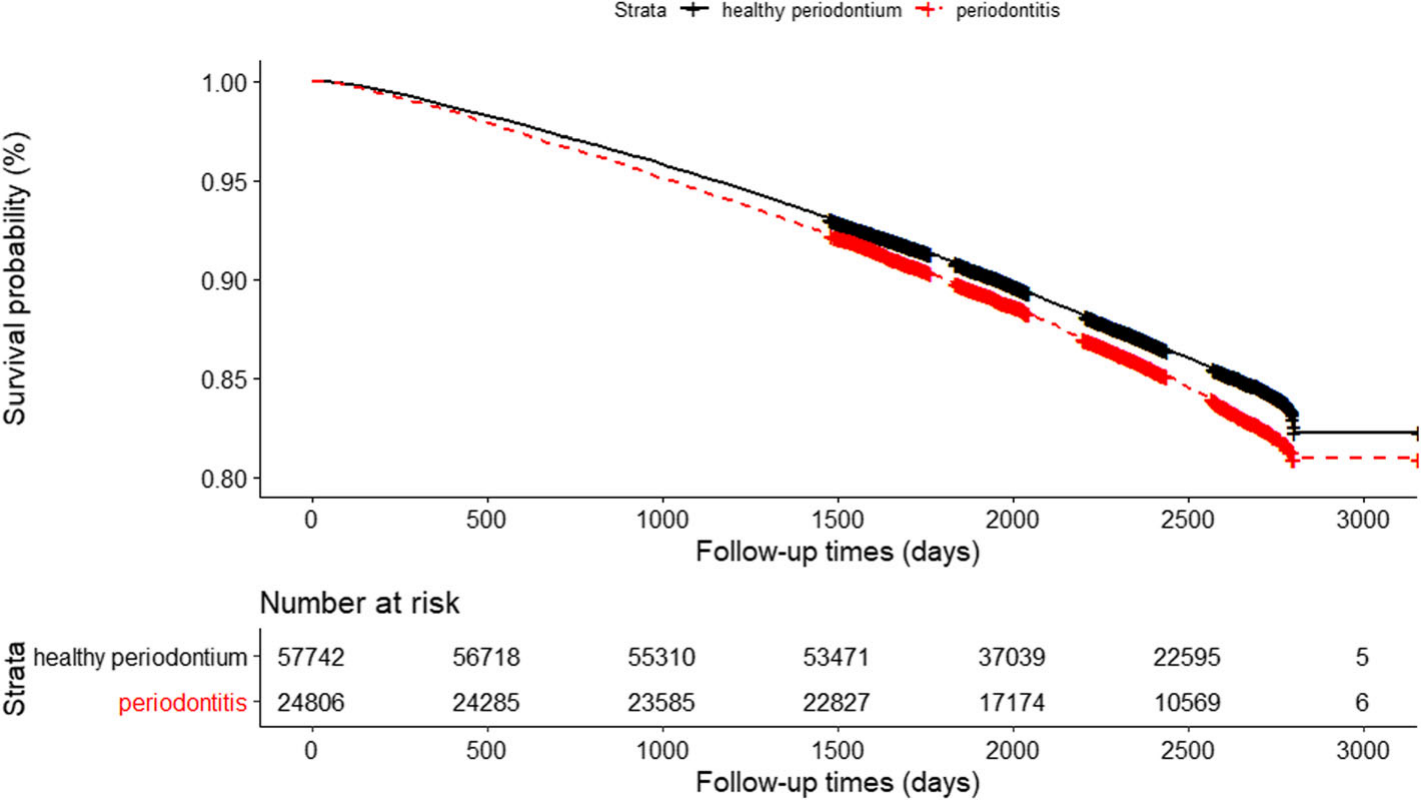
Kaplan-Meier graph of time to all-cause mortality by periodontal status

**Fig. 4:**
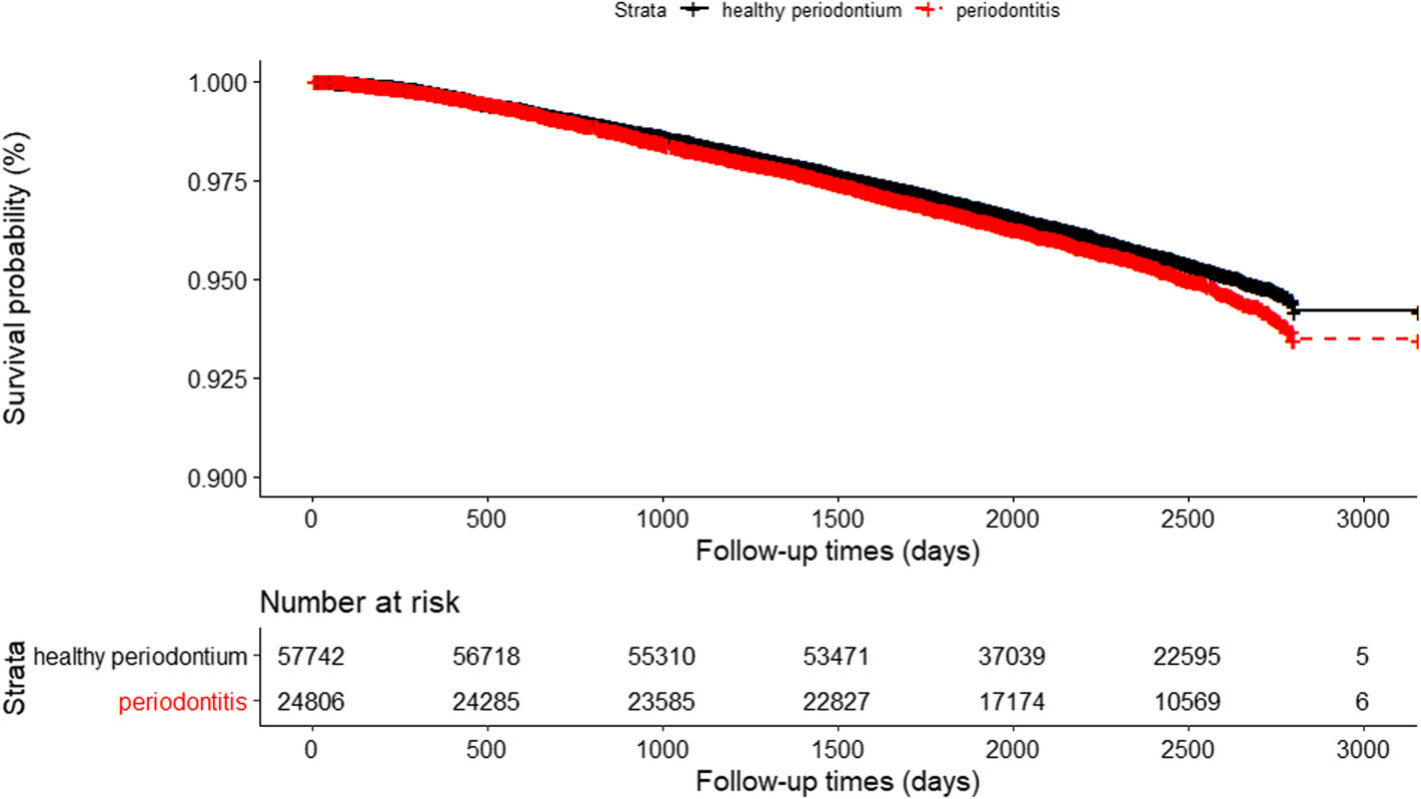
Kaplan-Meier graph of time to all-cancer mortality by periodontal status

At the midpoint of the study (1500 days), the survival probability of the periodontitis group was lower than that of the healthy periodontium group with regard to both all-cause mortality and all-cancer mortality (Figs. 3, 4). Of the 82,548 participants, 7460 of 57,742 (12.9%) in the healthy periodontium group and 3700 of 24,806 (14.9%) in the periodontitis group died by the end of the study. The estimated rate of overall survival at 3000 days in the Kaplan–Meier analysis was 80.9% (95% CI, 80.1 to 81.8) in the periodontitis group and 82.3% (95% CI, 81.3 to 83.3) in the healthy periodontium group. There were significant differences in the rates of survival between the two groups (*P* < 0.001).

Of the 82,548 participants, 2362 of 57,742 (4.1%) in the healthy periodontium group and 1153 of 24,806 (4.6%) in the periodontitis group died from cancer. The estimated rate of overall survival at 3000 days in the Kaplan–Meier analysis was 93.5% (95% CI, 92.9 to 94.1) in the periodontitis group and 94.2% (95% CI, 93.7 to 94.7) in the healthy periodontium group. There were significant differences in the rates of survival among the two groups (*P* = 0.004).

Table 2 shows the adjusted association of periodontitis with risk of all-cause mortality and all-cancer mortality in the baseline. After controlling for other covariates, participants with periodontitis had significantly higher hazard ratios (HRs) for all-cause mortality (HR = 1.077, 95% CI:1.027 to 1.130). A multivariate Cox proportional hazards model showed that being male (HR = 1.696, 95% CI:1.606 to 1.791), being elderly, and smoking (daily, HR = 1.253, 95% CI:1.126 to 1.394) were risk factors for all-cause mortality. Participants with a high education level (above 14 years of schooling, HR = 0.527, 95% CI: 0.480 to 0.579) had lower mortality. In regard to all-cancer mortality, after controlling for other covariates, hazard ratios (HRs) of all covariates had the same trend as that this result was not statistically significant for all-cancer mortality (HR = 1.036, 95% CI: 0.952 to 1.128).

**Table 2:** Hazard ratios of variables and mortality at baseline by multivariate Cox proportional hazards model

	All-cause mortality		All-cancer mortality	
	(*n* = 69,528, number of events = 7659)		(*n* = 69,528, number of events = 2483)	
	Hazard ratio	95% CI	Hazard ratio	95% CI
Periodontal condition				
healthy periodontium	reference		reference	
periodontitis	1.077*	1.027–1.130	1.036	0.952–1.128
Age	1.120*	1.117–1.124	1.078*	1.072–1.085
Marital status				
married living together	0.812*	0.772–0.853	0.965	0.881–1.058
other	reference		reference	
Education				
illiterate	reference		reference	
education 1–6 years	0.787*	0.724–0.856	0.920	0.781–1.083
education 7–14 years	0.613*	0.564–0.666	0.763*	0.648–0.897
education above 14 years	0.527*	0.480–0.579	0.626*	0.523–0.750
Sex				
female	reference		reference	
male	1.696*	1.606–1.791	1.719*	1.562–1.893
Smoking status				
no	reference		reference	
occasionally	1.576*	1.448–1.715	1.897*	1.653–2.177
daily	1.253*	1.126–1.394	1.655*	1.404–1.951

Abbreviations: *CI* Confidence interval

Variables included in the multivariate Cox proportional hazards model: age, marital status, education level, sex and smoking status

*: *p* < 0.05

Table 3 took annual health examinations results per participant into account. With regard to all-cause mortality and all-cancer mortality, there were significant associations with periodontitis (HR = 1.092, 95% CI: 1.038 to 1.149; HR = 1.114, 95% CI: 1.032 to 1.203) in multivariate Cox frailty models, after controlling for other covariates. Being male, having a low education level and being a smoker were risk factors for both all-cause mortality and all-cancer mortality when considering each visit.

**Table 3:** Hazard ratios of variables and mortality by multivariate Cox frailty model

	All-cause mortality		All-cancer mortality	
	(*n* = 245,768, number of events = 7652)		(*n* = 245,768, number of events = 6381)	
	Hazard ratio	95% CI	Hazard ratio	95% CI
Periodontal condition				
healthy periodontium	reference		reference	
periodontitis	1.092*	1.038–1.149	1.114*	1.032–1.203
Age	1.106*	1.103–1.110	1.002	0.992–1.013
Marital status				
married living together	0.782*	0.744–0.822	0.809*	0.683–0.959
other	reference		reference	
Education				
illiterate	reference		reference	
education 1–6 years	0.766*	0.704–0.833	0.740	0.538–1.017
education 7–14 years	0.592*	0.545–0.644	0.698*	0.510–0.954
education above 14 years	0.507*	0.462–0.557	0.573*	0.407–0.806
Sex				
female	reference		reference	
male	1.799*	1.704–1.899	2.712*	2.326–3.161
Smoking status				
no	reference		reference	
occasionally	1.412*	1.307–1.525	1.126	0.956–1.326
daily	1.753*	1.469–2.092	1.840*	1.455–2.326

Abbreviations: *CI* Confidence interval

Variables included in the multivariate Cox frailty model: age, marital status, education level, sex and smoking status

*: *p* < 0.05

### Association between periodontal status and risk of specific cancer mortality

Comparing mortality of lung cancer in the periodontitis group to the healthy periodontium group, the hazard ratio was 1.185 (95% CI: 1.027 to 1.368) after adjusting for age and sex in a multivariate Cox frailty model (Table S1). After adjusting for age and sex, the hazard ratio was 1.305 (95% CI: 0.856 to 1.989) for esophageal cancer, 1.019 (95% CI: 0.790 to 1.313) for pancreatic cancer, 0.960 (95% CI: 0.789 to 1.168) for liver and gallbladder cancer, 1.164 (95% CI: 0.952 to 1.423) for colorectal cancer, and 1.340 (95% CI: 1.019 to 1.762) for prostate cancer.

## Discussion

In this large elderly cohort, all-cause mortality and all-cancer mortality had positive association with periodontitis after adjusting for age, marital status, education level, sex and smoking status in Cox frailty models. Furthermore, in terms of specific cancers, lung and prostate cancer mortality were higher in the periodontitis group after adjusting for age and sex.

Bacterial biofilm invades the surrounding connective tissue of the gingiva, which may cause periodontitis. Direct or hematogenous spread of microorganisms increases the blood levels of inflammatory mediators, such as lipopolysaccharides and cytokines [32, 33]. The bacteria of dental plaque triggering systemic reactions are thought to lead to malignant transformation in a variety of tissues [34, 35]. Oral bacteria and inflammation may play a role in carcinogenesis [36]. The present findings show that dental plaque and gingival inflammation are associated with all-cancer mortality [33]. Furthermore, comorbidity and personal health behavior such as smoking status are important risk factors for periodontitis. Patients with periodontitis are more likely to have other systemic disease, an unhealthy lifestyle and low socioeconomic status, which also increases the mortality risk [37].

In our findings, lung cancer and prostate cancer had positive association with periodontitis after adjusting for age and sex. Previous studies were less consistent in finding a relationship between lung cancer and periodontitis. Hujoel et al. in a US-population-based cohort in which periodontal status was defined by standard dental examination, after adjusting for age category and sex, found a positive association between lung cancer mortality and periodontitis (odds ratio = 1.97 (95%CI:1.21–3.22)) [7].

In another study, when comparing lung cancer mortality rates among participants with and without periodontitis defined by whether they had periodontitis treatment procedure codes, the results indicated no significant difference in crude (1.31 (95%CI: 0.92–1.86)) and adjusted mortality rate ratios (1.20 (95%CI: 0.81–1.80)) which were adjusted for calendar time, age, sex, socio-economic status, number of teeth, dental treatments, oral health indices, need of periodontal treatment, and diabetes [9]. Arora et al., in a Swedish Twin Registry longitudinal study, suggested a positive association between incidence of prostate cancer and periodontitis classified by self-report after adjusting for potential confounders including sex, age, education, employment, number of siblings, smoking status, smoking status of partner, alcohol status, diabetes, and body mass index (odds ratio = 1.47 (95%CI:1.04–2.07)) [38]. Periodontal diseases can lead to tooth loss due to inflammatory conditions [39]. Hiraki et al., in a case control study, used tooth loss as an indicator, and a decreased number of teeth remaining was associated with a lower odds ratio for prostate cancer of 0.49 after adjusting for age, sex, smoking and drinking status, vegetable and fruit intake, BMI, and regular exercise [40].

However, the associations between some specific cancers and periodontitis are not consistent with previous research, including esophageal, pancreatic, colorectal, liver and gallbladder cancer. One of the reasons might be the low number of esophageal cancer cases (*n* = 64), which made inference uncertain. The lack of an association between periodontitis and other cancers may be due to characteristics of the participants. In some studies, smoking appears to be a confounding factor in risk of cancer, especially for cancers strongly linked to tobacco use such as lung cancer [7, 10, 41]. Hujoel et al. pointed out that when limited to non-smokers, no association between periodontitis defined by standard dental examination and lung cancer mortality was identified after adjusting for age, age squared, race, poverty index, education, and vitamin A and C; however, when limited to smokers, periodontitis was shown to be significantly associated with lung cancer [7]. For pancreatic cancer, the hazard ratio was not found to be substantially different after adjusting for smoking [10]. Michaud et al. have suggested that smoking is not likely to account for the excess risks at cancer sites such as pancreatic and kidney cancers [10].

Patients with diabetes have higher risk of developing periodontal disease. At the same time, periodontal disease may worsen the glucose control in diabetes patients. Several studies emphasize the impact of diabetes mellitus on subsequent risk of cancer [42–44]. In a Japanese hospital-based longitudinal study, a history of diabetes was associated with cancer risks for all sites for both males and females, controlling for age and potential confounders [45]. Patients with a past medical history of diabetes may have an increased incidence rate of specific cancers, notably pancreatic, liver, and colorectal cancer [44]. Previous studies usually took diabetes into account when exploring the relationship between periodontitis and mortality. One longitudinal population-based health survey showed a hazard ratio of 1.01 (95% CI: 1.002 to 1.01) for the association between the extent of clinical attachment level ≥ 3 mm and all-cause mortality after adjusting for age, sex, household income, years of education, body mass index, smoking, physical activity, and dental checkup. However, the findings did not indicate additive interaction of periodontal destruction and diabetes regarding all-cause mortality. Kebede et al. pointed out that despite the reciprocal relationship between periodontal destruction and diabetes, they may be independent risk factors for all-cause mortality [46].

Previous studies have proposed a mechanism by which vitamin D decreases the risk of periodontal disease and decreases the risk of several cancers [47]. Individuals with less vitamin A and C are more likely to have periodontitis [7]. Individuals with less fruit and vegetable intake have increased risk of cancer of specific sites such as oral cancer and gastric cancer [48]. Less intake of fruit and vegetables may be due to tooth loss resulting from advanced periodontal disease [39].

Our limitations include, first, the fact that the models were not adjusted for comorbidities such as respiratory disease, because the dataset didn’t record respiratory function and respiratory diseases. Besides, physical activity was missing in 2005 and 2012, so we couldn’t include this variable for analysis. Second, the outcome and the exposure could be affected by unmeasured confounding factors. And third, confounders were considered, but measuring bias might exist in self-reported data such as smoking status, alcohol consumption and daily fruit and vegetable intake. Furthermore, the quantity of alcohol consumption was not assessed. The results for alcohol consumption, daily fruit and vegetable intake and other comorbidities including diabetes, hypertension and cardiovascular disease are shown in the supplement (Table S2 and Table S3). Finally, the periodontal status in this study was diagnosed by dentists, not self-reported by the participants. Because our data are secondary data, we don’t have information about the consistency of periodontal health assessment, in the form of agreement between dentists.

Within the same age group as our study, the mortality rates in Taipei and nationally in 2012 were 0.62 and 0.67% respectively. In this study the mortality rate in 2012 was 0.59%, which was slightly lower than the national mortality rate in the same period. The common reasons for non-participation among the elderly were unsuitable timing or location of the health examinations, lacking time, or feeling too sick to participate [49, 50]. Because the health examinations in our study were performed by Taipei-contracted hospitals in each administrative division in Taipei, citizens had easy access. Nonparticipants in our study might be those with poorer health. Moreover, Taipei is a highly urbanized city, and has a mortality rate lower than the national mortality rate. In urbanized areas, age-standardized mortality rates were lower than those in suburban and rural areas [51]. Our estimates of hazard ratios might be slightly low.

Strengths of our study are the large sample, and the fact that these participants were followed across time. Over 30% of the sample were diagnosed as having periodontitis at the baseline. Regular health screening seems important due to the high prevalence of this disease. Health screening allows early identification of chronic non-communicable diseases and its risk factors, which easily happen in the elderly. Health screening can lead to the reduction of related complications as well as mortality [52]. Screening for health problems that can emerge in later life could reduce the burden of disease. Regular oral examination can detect the signs and symptoms of oral disease early, in particular dental caries and periodontal disease [53]. Lowering periodontitis prevalence may reduce the medical expenditures of health care systems [14, 54].

## Conclusions

Being male, having a low education level, and being a smoker were risk factors for mortality in this retrospective elderly community cohort study. Our study findings showed mixed evidence that periodontal disease is associated with all-cause, all-cancer and specific-cancer mortality including lung and prostate cancer. It is suggested that governments enact policies emphasizing health screening for early disease detection and educating people on the importance of regular health examinations.

## Supplementary information

**Additional file 1: Table S1.** Hazard ratios of different kinds of mortality. **Table S2.** Hazard ratios of variables and mortality by multivariate Cox proportional hazards model (baseline). **Table S3.** Hazard ratios of variables and mortality by multivariate Cox frailty model

## Data Availability

The raw data are confidential and cannot readily be shared. Researchers need to obtain permission from the Institutional Review Board and apply for access to the data from the Department of Health, Taipei City Government.

## Abbreviations

CI: Confidence interval
CPI: Community periodontal index
HRs: Hazard ratios (HRs)
