# Supplementary material for: Association between periodontitis and all-cause and cancer mortality: retrospective elderly community cohort study
Source: BMC Oral Health. 2020 Jun 9;20:168. doi: 10.1186/s12903-020-01156-w (PMC7285774; doi:10.1186/s12903-020-01156-w)
Supplement: Supplementary file 1 — Additional file 1: Table S1. Hazard ratios of different kinds of mortality. Table S2. Hazard ratios of variables and mortality by multivariate Cox proportional hazards model (baseline). Table S3. Hazard ratios of variables and mortality by multivariate Cox frailty model [file 12903_2020_1156_MOESM1_ESM.docx]

**Table S1. Hazard ratios of different kinds of mortality**

| Kinds of mortality | Number of events | Hazard ratio | Lower CI | Upper CI |
| --- | --- | --- | --- | --- |
| All-cause mortality | 11160 | 1.150 | 1.104 | 1.198 |
| All cancers | 3515 | 1.129 | 1.050 | 1.213 |
| Lung cancer | 916 | 1.185 | 1.027 | 1.368 |
| Esophageal cancer | 64 | 1.305 | 0.856 | 1.989 |
| Pancreatic cancer | 208 | 1.019 | 0.790 | 1.313 |
| Liver and gallbladder cancer | 495 | 0.960 | 0.789 | 1.168 |
| Colorectal cancer | 431 | 1.164 | 0.952 | 1.423 |
| Prostate cancer | 182 | 1.340 | 1.019 | 1.762 |

Abbreviations: CI=confidence interval.

multivariate Cox frailty model adjusted for age and sex.

**Table S2. Hazard ratios of variables and mortality by multivariate Cox proportional hazards model (baseline)**

|  | All-cause mortality | | |  |  |  | All-cancer mortality | | |  |  |
| --- | --- | --- | --- | --- | --- | --- | --- | --- | --- | --- | --- |
|  | (n= 69269,  number of events= 7632) | | | | |  | (n= 69269,  number of events= 2477) | | | | |
|  | Hazard ratio |  | 95% CI | |  |  | Hazard ratio |  | 95% CI |  |  |
| Periodontal condition |  |  |  |  |  |  |  |  |  |  |  |
| healthy periodontium | reference |  |  |  |  |  | reference |  |  |  |  |
| periodontitis | 1.076 |  | 1.026 | - | 1.129 |  | 1.034 |  | 0.950 | - | 1.125 |
| Age | 1.119 |  | 1.115 | - | 1.123 |  | 1.078 |  | 1.071 | - | 1.084 |
| Marital status |  |  |  |  |  |  |  |  |  |  |  |
| married living together | 0.826 |  | 0.785 | - | 0.868 |  | 0.974 |  | 0.889 | - | 1.068 |
| other | reference |  |  |  |  |  | reference |  |  |  |  |
| Education |  |  |  |  |  |  |  |  |  |  |  |
| illiterate | reference |  |  |  |  |  | reference |  |  |  |  |
| education 1-6 years | 0.804 |  | 0.740 | - | 0.875 |  | 0.951 |  | 0.806 | - | 1.122 |
| education 7-14 years | 0.644 |  | 0.593 | - | 0.701 |  | 0.800 |  | 0.679 | - | 0.943 |
| education above 14 years | 0.562 |  | 0.512 | - | 0.618 |  | 0.661 |  | 0.551 | - | 0.793 |
| Sex |  |  |  |  |  |  |  |  |  |  |  |
| female | reference |  |  |  |  |  | reference |  |  |  |  |
| male | 1.769 |  | 1.674 | - | 1.870 |  | 1.716 |  | 1.555 | - | 1.894 |
| Smoking status |  |  |  |  |  |  |  |  |  |  |  |
| no | reference |  |  |  |  |  | reference |  |  |  |  |
| occasionally | 1.652 |  | 1.516 | - | 1.801 |  | 1.919 |  | 1.667 | - | 2.208 |
| daily | 1.338 |  | 1.202 | - | 1.491 |  | 1.683 |  | 1.425 | - | 1.987 |
| Eating fruits and vegetables |  |  |  |  |  |  |  |  |  |  |  |
| no | reference |  |  |  |  |  | reference |  |  |  |  |
| yes | 0.790 |  | 0.749 | - | 0.832 |  | 0.864 |  | 0.787 | - | 0.948 |
| Diabetes |  |  |  |  |  |  |  |  |  |  |  |
| no | reference |  |  |  |  |  | reference |  |  |  |  |
| yes | 1.329 |  | 1.269 | - | 1.392 |  | 1.206 |  | 1.111 | - | 1.309 |
| Alcohol consumption |  |  |  |  |  |  |  |  |  |  |  |
| no | reference |  |  |  |  |  | reference |  |  |  |  |
| yes | 0.711 |  | 0.667 | - | 0.759 |  | 0.899 |  | 0.810 | - | 0.997 |
| unfilled | 0.745 |  | 0.240 | - | 2.311 |  | 0.887 |  | 0.125 | - | 6.307 |
| Hypertension |  |  |  |  |  |  |  |  |  |  |  |
| no | reference |  |  |  |  |  | reference |  |  |  |  |
| yes | 1.099 |  | 1.049 | - | 1.152 |  | 1.026 |  | 0.946 | - | 1.112 |
| Cardiovascular disease |  |  |  |  |  |  |  |  |  |  |  |
| no | reference |  |  |  |  |  | reference |  |  |  |  |
| yes | 1.004 |  | 0.958 | - | 1.053 |  | 0.922 |  | 0.849 | - | 1.001 |

Abbreviations: CI=confidence interval.

Variables included in the multivariate Cox proportional hazards model: age, marital status, education level, sex, smoking status, eating fruits and vegetables, diabetes, alcohol consumption (self-reported alcohol consumption in the past 6 months), hypertension (history of cardiovascular disease or long-term medication, or abnormal biochemical data including total cholesterol, triglyceride and high-density lipoprotein) and cardiovascular disease (history of hypertension or long-term medication, or high blood pressure).

**Table S3. Hazard ratios of variables and mortality by multivariate Cox frailty model**

|  | All-cause mortality | | |  |  |  | All-cancer mortality | | |  |  |
| --- | --- | --- | --- | --- | --- | --- | --- | --- | --- | --- | --- |
|  | (n= 233918,  number of events= 7532) | | | | |  | (n= 233918,  number of events= 6341) | | | | |
|  | Hazard ratio |  | 95% CI | |  |  | Hazard ratio |  | 95% CI |  |  |
| Periodontal condition |  |  |  |  |  |  |  |  |  |  |  |
| healthy periodontium | reference |  |  |  |  |  | reference |  |  |  |  |
| periodontitis | 1.100 |  | 1.045 | - | 1.157 |  | 1.113 |  | 1.030 | - | 1.202 |
| Age | 1.105 |  | 1.101 | - | 1.108 |  | 1.005 |  | 0.994 | - | 1.015 |
| Marital status |  |  |  |  |  |  |  |  |  |  |  |
| married living together | 0.796 |  | 0.757 | - | 0.837 |  | 0.799 |  | 0.674 | - | 0.949 |
| other | reference |  |  |  |  |  | reference |  |  |  |  |
| Education |  |  |  |  |  |  |  |  |  |  |  |
| illiterate | reference |  |  |  |  |  | reference |  |  |  |  |
| education 1-6 years | 0.791 |  | 0.727 | - | 0.861 |  | 0.741 |  | 0.539 | - | 1.020 |
| education 7-14 years | 0.630 |  | 0.579 | - | 0.685 |  | 0.698 |  | 0.510 | - | 0.956 |
| education above 14 years | 0.548 |  | 0.499 | - | 0.603 |  | 0.566 |  | 0.402 | - | 0.797 |
| Sex |  |  |  |  |  |  |  |  |  |  |  |
| female | reference |  |  |  |  |  | reference |  |  |  |  |
| male | 1.905 |  | 1.802 | - | 2.014 |  | 2.733 |  | 2.342 | - | 3.190 |
| Smoking status |  |  |  |  |  |  |  |  |  |  |  |
| no | reference |  |  |  |  |  | reference |  |  |  |  |
| occasionally | 1.507 |  | 1.394 | - | 1.630 |  | 1.131 |  | 0.959 | - | 1.334 |
| daily | 1.829 |  | 1.531 | - | 2.186 |  | 1.840 |  | 1.454 | - | 2.329 |
| Eating fruits and vegetables |  |  |  |  |  |  |  |  |  |  |  |
| no | reference |  |  |  |  |  | reference |  |  |  |  |
| yes | 0.802 |  | 0.762 | - | 0.843 |  | 1.098 |  | 1.003 | - | 1.202 |
| Diabetes |  |  |  |  |  |  |  |  |  |  |  |
| no | reference |  |  |  |  |  | reference |  |  |  |  |
| yes | 1.221 |  | 1.166 | - | 1.278 |  | 0.948 |  | 0.867 | - | 1.037 |
| Alcohol consumption |  |  |  |  |  |  |  |  |  |  |  |
| no | reference |  |  |  |  |  | reference |  |  |  |  |
| yes | 0.606 |  | 0.580 | - | 0.664 |  | 0.960 |  | 0.858 | - | 1.073 |
| unfilled | 1.020 |  | 0.458 | - | 2.271 |  | 0.982 |  | 0.118 | - | 8.162 |
| Hypertension |  |  |  |  |  |  |  |  |  |  |  |
| no | reference |  |  |  |  |  | reference |  |  |  |  |
| yes | 0.999 |  | 0.953 | - | 1.048 |  | 0.929 |  | 0.851 | - | 1.014 |
| Cardiovascular disease |  |  |  |  |  |  |  |  |  |  |  |
| no | reference |  |  |  |  |  | reference |  |  |  |  |
| yes | 1.013 |  | 0.966 | - | 1.602 |  | 0.984 |  | 0.908 | - | 1.066 |

Abbreviations: CI=confidence interval.

Variables included in the multivariate Cox proportional hazards model: age, marital status, education level, sex, smoking status, eating fruits and vegetables, diabetes, alcohol consumption (self-reported alcohol consumption in the past 6 months), hypertension (history of cardiovascular disease or long-term medication, or abnormal biochemical data including total cholesterol, triglyceride and high-density lipoprotein) and cardiovascular disease (history of hypertension or long-term medication, or high blood pressure).
